# Divergent Leaf and Stem Traits but Similar Flammability in Two Contrasting Tropical *Grevillea* Species

**DOI:** 10.3390/biology15212031

**Published:** 2026-11-07

**Authors:** David Yue Phin Tng, Shea Elsie Issendorf, Elizabeth Joanne Karatzas, Hung Minh Pham, Deborah Mattos Guimaraes Apgaua

**Affiliations:** 1 Centre for Rainforest Studies, School for Field Studies, Barrine, QLD 4884, Australia; 2 College of Food, Agriculture and Natural Resources, The University of Minnesota, Minneapolis, MN 55455, USA; 3 College of Natural Sciences, The University of Massachusetts Amherst, Amherst, MA 01003, USA; elliekaratzas@gmail.com; 4 Department of Biology, Denison University, Granville, OH 43023, USA; pham_h3@denison.edu

**Keywords:** congeneric species, ecological strategies, hydraulic architecture, functional traits, trait variation, Proteaceae

## Abstract

Climate change has led to more unpredictable and in many cases more extreme environmental stress on both forests and savannas, which occur as a vegetation mosaic in many tropical regions. It is therefore important to understand the comparative ecological strategies of plants in these disparate habitats. Studying a pair of congeneric *Grevillea* (Proteaceae) in the forest (*Grevillea baileyana)* and in the savanna (*Grevillea glauca*), which do not overlap in their habitat, we examined 20 functional stem and leaf traits, including less-studied anatomical and flammability traits. We found that the forest *Grevillea* had a higher leaf area, stomatal density, and vessel area, while the savanna species exhibited denser wood, more parenchyma, higher leaf mass per unit area, and thicker bark (*p* < 0.001 for most trait comparisons). However, we found that the forest species was equally flammable as the savanna species, with the latter only having a higher maximum burn temperature. Overall, our results are consistent with the forest species having more acquisitive and the savanna species having more resource-conservative trait strategies. Further examination of intraspecific trait variation will also aid in understanding how these species may respond to the effects of climatic change in their respective habitats.

## Simple Summary

Tropical rainforests and savannas experience very different environmental conditions, particularly in water availability and exposure to fire. Understanding plant ecological strategies in contrasting environments can help us predict how they may respond to future environmental change. We compared 20 leaf, stem, anatomical, and flammability traits in two closely related Australian tree species: *Grevillea baileyana*, which occurs in rainforest, and *Grevillea glauca*, which occurs in savanna. The two species differed significantly in many of their traits. The rainforest species generally had traits associated with greater resource use and water transport, whereas the savanna species showed traits associated with conserving water and coping with a drier environment, including thicker leaves and bark. Surprisingly, the two species differed little in the flammability traits we measured, despite the much greater exposure to fire in savannas. Our findings show that closely related species can develop markedly different ecological strategies in contrasting environments, while some traits may remain similar. Studying such species pairs can improve our understanding of how plants may respond to changing climate, drought, and fire regimes.

## 1. Introduction

Global climate change is driving increased temperatures, prolonged droughts and modified fire regimes at regional scales in many countries, presenting formidable challenges to various terrestrial ecosystems and the flora and fauna that inhabit these environments [1]. The tropical rainforest biomes, where plants are adapted to stable environments with infrequent perturbations and high water availability [2,3], are expected to be particularly at risk, as they may not possess ecological strategies that help them deal with prolonged drought conditions or increased fire exposure [4].

Where tropical rainforests have been severely disturbed by fires or have experienced massive tree die-back due to drought, a biome switch could be triggered [5,6], leading to a rapid simplification of their vegetation structure and changes in biological composition. Even savanna trees, which are often more resilient to disturbances such as fire and drought [7,8], may still face an increased susceptibility to mortality if they are placed under excessive stress due to warmer temperatures or prolonged drought [8,9]. Contrastingly, some regions in the world may experience the opposite, where a wetting trend, or reduction in fire regimes and increased atmospheric CO_2_, may be leading to vegetation thickening and canopy closure over what were once open savanna areas [5,10,11].

Savanna plants, by virtue of their adaptations to open high-light environments, may not be able to thrive in increasingly shaded and wet environments. Underpinning the narrative on these two opposite ecological scenarios is the fundamental question of how the ecological strategies of plants in these two environments differ. Comparing the functional traits of forest plants with those of savanna forests would establish a robust understanding of species- or habitat-associated differences in ecological strategies of plants [12,13].

Functional traits represent an increasingly popular way of studying ecological strategies [14,15]. These traits specifically refer to morphological, physiological or anatomical features that serve as useful and standardized parameters for studying plants growing in different environments or changes in ecosystem function [14,15,16]. Leaf and stem morphological traits such as leaf area, leaf mass per unit area, wood density, Huber value (sapwood-to-leaf area ratios), and tree heights are commonly measured traits because they define plant ecological strategies along different resource gradients [17,18]. For example, leaf area and leaf mass per unit area measure the organ surface area available for water transpiration and reflect the investment in structural support or protective tissues in leaves [17]. A key stem trait, wood density, is also often associated positively with drought tolerance [19], and thus woody plants in wet habitats generally have less dense wood than species from drier habitats.

Another class of functional traits that are pertinent to understanding the differences between rainforest and savanna species relates to flammability. Yet, there have been very few studies that have directly compared plant flammability differences between forest and savanna plants [20], and these could have important real-world implications. For example, if forests are as flammable as savanna species, this has implications for fire management and public safety in forested regions subjected to prolonged drought. One simple way to compare flammability would be to obtain measures of maximum burn temperatures, burn duration and burnt biomass of field-collected branch samples on a stove top [21,22]. Since savannas are fire-prone and forests are fire-sensitive environments, it is reasonable to expect that savanna species will consistently exhibit higher flammability measures than forest species.

While functional traits are often perceived as “soft” traits that are proxies for harder-to-measure physiological traits, not all functional traits are straightforward to measure. A class of functional traits that have been gaining more attention in the last two decades is anatomical traits [23,24,25,26]. Various leaf traits such as stomatal densities and wood anatomical traits such as stem vessel sizes or densities make up the hydraulic architecture of a species and can be useful proxy for plant physiological processes at various levels of biological organization from tissue, organ level and whole plants [27]. A case could therefore be made for establishing the concept of an anatomical economic spectrum [25], and over the last decade, ecologists studying plant function have increasingly incorporated anatomical traits to examine unique trait-associations within or trait-tradeoffs across species [26,28,29,30,31,32], and to investigate plant–environment relationships [33,34].

Our objective is to conduct an exploratory study of how rainforest and savanna trees differ in their ecological strategies by studying functional traits in a same-genus (congeneric) pair of species, with one species in each study habitat, e.g., [26,35,36]. Such an approach can provide clear and more robust comparisons between the two groups when there is a large amount of trait variation among genera [37,38]. More importantly, the plants being compared are phylogenetically independent, and therefore we can assume any variations in functional traits between the two closely related trees are habitat-associated rather than from having a different ancestry [39].

We therefore hypothesize that congeneric forest and savanna species will possess drastically contrasting trait behaviors, with the forest species exhibiting trait strategies that are more acquisitive in nature, while the traits of savanna species would be reflective of resource conservation. For ease of discussion, we categorize these traits into four different categories: the leaf economics spectrum [17], the stem economics spectrum [18,24], shoot flammability [20] and leaf and wood anatomical traits related to plant hydraulic architecture [25,32,40]. These translate to a broad set of premises and predictions on how plants from contrasting biomes will differ in their trait behavior and composition (Table 1).

## 2. Materials and Methods

### 2.1. Study Region

Our study sites are situated within rainforest and savanna habitats in North Queensland, Australia. The rainforest site is within the campus grounds of the Centre for Rainforest Studies (CRS), School for Field Studies (17°12′ S, 145°40′45″ E; 759 m a.s.l.), which is in Danbulla on the eastern edge of the Atherton Tablelands. The vegetation surrounding the Centre is upland tropical rainforests locally classified as simple notophyll vine forests [53,54]. This property was previously cleared for agriculture and dairy farming but has since been revegetated (Queensland Government). The closest reliable weather records for the site are from Lake Barrine, which is 9.8 km southwest of the study site, where the mean annual rainfall in this region is approximately 2500 mm and mean annual temperatures range from 15.5 °C to 25 °C per [53].

The savanna study site is located 26 km northwest of Danbulla within private property at Granite Gorge, Mareeba, Queensland (17°02′ S, 145°20′30″ E; 400 m a.s.l.). The savanna vegetation at the site is dominated by open canopies of *Corymbia clarksoniana* and *Eucalyptus crebra* (Myrtaceae), with a high cover of grasses and forbs in the ground layer. This site experiences a drier climate with an average rainfall of 912 mm per year, and annual minimum and maximum temperatures of 16.1 °C and 28.8 °C [55].

### 2.2. Target Species

To examine trait divergence, we studied two congeneric (same-genus) species of *Grevillea* (Proteaceae). The Proteaceae is a Gondwanan family with a long evolutionary history in the southern continents stretching back 132 million years, with some rainforest species being basal lineages, although rainforest species also occur throughout the phylogeny among genera largely represented by taxa that occupy open environments [56]. The cyclical phases of wet and dry periods throughout the long evolutionary history of Proteaceae have resulted in various events where members of the Proteaceae radiated from closed forests out to open environments, with the result that the majority of known Proteaceae are now plants of open environments [56].

Our target genus, *Grevillea*, is represented by members in both forest and savanna biomes. The rainforest representative, *Grevillea baileyana* McGill, is a tree found in lowland to upland rainforests of Australia and New Guinea. *G. baileyana* grows up to 30 m tall and has gray scaly bark. The leaves are strongly heteroblastic, meaning that they exhibit extreme variation in morphology during plant development [57]. Therefore, younger leaves and leaves positioned on lower branches are typically lobed or pinnatifid, while mature leaves are elliptic. The leaf undersides are brown and rusty and contrast with the dark green appearance on the top side [57].

The savanna representative, *Grevillea glauca* Banks & Solander ex Knight, is a widespread species found on various substrates in seasonally dry open forests, woodlands, savannas, and also occasionally in coastal dune heaths of northeastern Queensland and Papua New Guinea [58]. The species grows up to 10 m tall but can be reproductively mature and flower at a height of 2 m. *G. glauca* has furrowed dark brown to black bark and has leaves that are narrowly ovate to elliptic, which are only weakly heteroblastic. Importantly, the leaves differ from those of *G. baileyana* in being isobilateral, with stomata located on both the front and back of the leaves (pers. obs.).

### 2.3. Sample Collection

We designed our sampling regime to address our two related objectives to examine inter- and intraspecific trait variation in congeneric *Grevillea* species. As we were sampling at the population level, we wanted to obtain sufficient replication for robust comparisons of trait variability at both inter- and intraspecific levels. For this reason, we sampled a high number of replicates (20 replicate individuals) for each of our target species, and collected the same panel of traits (Table 1) for each replicate individual. This sampling regime is two- to four-times the recommended number of samples for most of the functional traits relevant in our study and listed in the trait handbook by Pérez-Harguindeguy et al. [59]. All sampling was conducted within two weeks, between late March and early April 2024, and during the wet season.

To obtain sufficient material for all the traits to be measured, we collected 1.5 m long branches from each replicate individual and subsampled leaves or wood for specific traits. For the rainforest species, *G. baileyana*, we collected samples from the outer crown using a fully extended 5 m long ladder and a 2 m extension pruning pole, allowing us to collect outer crown samples at heights of at least 5 m from the ground to limit collection to the simple elliptic leaves and to avoid sampling young, lobed leaves. Individual replicates of *G. baileyana* ranged from 6 to 15 m tall and had diameters at 1.3 m height (diameter at breast height: DBH) ranging from 6 to 25 cm. For the savanna species, *G. glauca*, our replicate individuals had a comparable DBH range (5 to 24 cm) but were inherently shorter (4 to 10 m in height), and so we used a shorter 1.5 m ladder in conjunction with pole pruners to assist in branch collection.

Each collected branch was then partitioned into one 20 cm and one 100 cm long sample when measuring from the proximal end of a branch. Both samples were ensured to be intact or unbroken and missing few to no leaves, but the 20 cm sample was the most complete, fully extended, with the fewest scar tissues, and non-flowering or -fruiting section of each collected sample, which will be used for calculation of Huber value and leaf area. Both were ensured to be intact or unbroken and missing few to no leaves.

Chlorophyll content was measured in the field on three fully expanded leaves on each branch using an atLEAF CHL PLUS chlorophyll meter (FT Green LLC, Wilmington, DE, USA), a handheld device that measures relative chlorophyll content in a non-destructive manner.

We collected bark thickness (BT: mm) at 1.3 m height using a bark gauge. The BT measurement process was conducted in each of the cardinal directions. The height of each tree was also approximated in meters using a constant reference.

### 2.4. Leaf Traits

To measure leaf area, the leaves from each 20 cm subsample were pressed under a glass pane and photographed alongside a 2 × 2 cm scale. The average leaf area (LA: mm^2^) was measured in pixel units from the leaf photographs using the magic wand function in the ImageJ software (v. 1.54c) and converted to mm^2^ by calibration with the 2 × 2 cm scale in each photograph. The Huber value was calculated using the ratio of the total leaf area of all the leaves on a 20 cm branch and the sapwood cross-section area [60].

To obtain leaf mass per unit area (LMA: g m^−2^), leaf dry matter content (LDMC: mg g^−1^), leaf thickness (LT: mm) and leaf volume (LV: mm^3^), we excised five intact leaves from the 100 cm main sample of each individual. These bulked leaves were first weighed using a weighing scale, and then their combined areas were obtained using the same method as described earlier for leaves on the 20 cm branch subsample. We then dried these leaves in an oven at 75 °C for seven days and then obtained their dry mass. LMA was then calculated by dividing the combined dry mass by the summed LA of each sample, while LDMC was derived by dividing the combined dry mass by the fresh mass [59]. The same five leaves were then measured with a digital micrometer (mm) to measure leaf thickness (LT: mm). To calculate leaf volume (LV), we multiplied the leaf area by the leaf thickness of each of the five leaves and obtained the averages of these values.

### 2.5. Stem Traits

To obtain wood density (WD: g cm^−3^), we used the water displacement method based on the Archimedes principle [59]. This involved first cutting two 5 cm wood subsamples from the proximal end of the 100 cm stem sample, shaving off the bark. These subsamples were then mounted on a needle fixed to a retort stand and slowly submerged beneath the water in a beaker placed on a laboratory balance. In a few instances where small bubbles formed on the subsample surface, we gently nudged the subsample to dislodge the bubbles to the surface. We then converted the recorded mass change (g) to volume by dividing it by the density of water at 20 °C (0.998 g cm^−3^). Following displacement measurements, the wood subsamples were dried in an oven at 115 °C to a constant weight and wood density was calculated by dividing the oven-dry mass of these samples by their volume.

We obtained the bark thickness and diameter at breast height (1.3 m height) or stem diameter (DBH: cm) of each individual tree. On each tree, we measured bark thickness (BT: mm) at the four cardinal points of the trunk at 1.3 m height using a standard bark gauge (Haglof, Barktax, Långsele, Sweden). Measured this way, bark thickness includes the cambium in most instances, but since cambium is relatively thin (<0.5 mm), we deemed this to have no substantial effect on our sampling [46]. Relative bark thickness was then calculated by dividing the average bark thickness by the DBH (inclusive of the bark) of each tree [46].

### 2.6. Anatomical Traits

We obtained stomatal density (mm^−2^) from leaf lamina subsamples. Obtaining cuticle impressions using the nail polish method was not suitable for our species due to the presence of densely matted leaf trichomes in both species. We therefore used a different approach where we physically peeled off the cuticle to be able to better examine the stomata. To accomplish this, we lightly brushed the midleaf lamina on the abaxial leaf surface using 600-grade sandpaper to remove some trichomes. We then cut out 2 cm × 2 cm square portions and soaked these leaf squares in Petri dishes containing 75% sodium hypochlorite until they were completely discolored. The leaf squares were then rinsed with water, placed onto a slide, and the cuticle was peeled off from the leaf square with fine forceps and placed onto the slide adjacent to the leaf square in a manner resembling the flipping of a page in a book. In this manner, the hairs of the cuticle peel are resting on the slide, and when stained with toluidine blue, the hair bases stand out in blue, contrasting with stomata that do not stain. Due to the isobilateral leaf nature of *G. glauca*, stomata were present in equal numbers on both sides of the leaf, so we used cuticle peels from one or the other surfaces of the leaf. Pictures of each processed subsample were taken at 40× magnification and then the stomata in each picture were counted. The image area was calculated using a calibration slide and the ImageJ software, and the number of stomata was divided by the image area to calculate stomatal density.

For stem anatomy, we obtained stem transverse sections from the proximal end of each 20 cm sample using the GSL1 portable microtome (Schenkung Dapples, Zurich, Switzerland) [61]. The stem sections were then dyed with Toluidine blue and mounted on a slide with glycerine jelly. Using an Optico GT Series Digital Microscope Camera (5MP, USB2.0) (Tucsen Photonics Co., Ltd., Fuzhou, China), we photographed the stem sections at 100× magnification, and these digital images were processed in the ImageJ software. From these digital images, we measured six different wood anatomy traits: vessel area (VA), vessel density, vessel grouping index (VGI), vessel fraction, parenchyma fraction, and fiber fraction.

Vessel area (VA: μm^2^) was measured using ImageJ based on the stomatal density. For each sample, we took two microphotographs and measured the area of 25 random vessels in each photo using length and breadth measurements. For the area of elliptic shapes such as these vessels, we used the formula A = πab, where A is the area, and a and b are the two diameter (μm) measurements of each vessel. The vessel areas of the 25 random vessels were then averaged.

Vessel grouping index (VGI) was calculated using the formula: VGI = N_Vessels_/N_groupings_. A VGI value of 1 indicates that all vessels are solitary, and the higher the VGI, the more groupings there are in a cross-sectional image. Vessel groupings (solitary and multiple vessel groupings) were counted based on the technique demonstrated by Scholz et al. [40]. The area of measurement was considered to be the whole photo, calculated using the image area method as in the stomatal density section. Vessel density (V_den_: mm^−2^) was calculated by dividing the number of vessels in each image by the image area. In addition, we calculated the cavitation vulnerability index (CVI) by dividing the average vessel diameter by the vessel density [50].

Vessel, parenchyma, and fiber fractions were calculated by overlaying a 10 × 10 grid with dimensions of 600 microns × 600 microns over a 10× magnification image of each stem transverse section. We then classified the point on the microphotograph at each of the 100 intersections as “vessel”, “parenchyma”, or “fiber cell” in the overlaid grid. Vessel, parenchyma, and fiber fractions were then expressed as a percentage of each of those tissue types out of the 100 intersections.

### 2.7. Flammability Measures

To obtain shoot flammability measures, we used a 70 cm branch subsample that was cut from each 100 cm sample of each individual. These samples were collected in the morning, and each 70 cm branch was left to equilibrate at the laboratory shed overnight to match the moisture content of the sample to the ignition source [21] before burning trials were undertaken. Burning trials were conducted following an established protocol by Potts et al. [22] and Ortiz et al. [20]. This method has the added benefit of accounting for the influence of plant architecture on flammability [22,62,63,64]. Prior to burning samples, the heating element in the flammability apparatus was turned on and warmed up to an average temperature of 100–150 °C, which we measured at three equidistantly spaced locations on the grill surface using a set of thermocouples. These thermocouples consisted of three 2-input thermocouple thermometers (−50–1300 °C), fitted with a K-type thermocouple probe per device (−5–250 °C [±2.5 °C]). We placed the branch on a stove for 120 s as part of a preheating step that simulates a fire front, after which a blowtorch flame was applied to the distal end of the branch for 10 s to provide an ignition source [20]. Once the branch ignited, the duration of burn (burn time: seconds) was recorded using a stopwatch, and the maximum burn temperature (Max. temp.: °C) was measured using a handheld FLUKE 572–2 infrared thermometer (Fluke Corporation, Everett, WA, USA). After the flames extinguished by itself, we estimated the percent of biomass consumed (burnt biomass: %) of the branch. All samples burned, so ignition frequency was 100% for both species.

### 2.8. Data Analysis

To evaluate functional trait divergence and flammability constraints between our congeneric study species, we utilized an Analysis of Covariance (ANCOVA) framework executed via Generalized Linear Models (GLMs). For each model, the individual plant trait was designated as the response variable, habitat type (Rainforest vs. Savanna) as the fixed main effect, and tree diameter (DBH) as a continuous fixed covariate to statistically control for ontogenetic size effects. Sampling was structured with 20 individual trees per species (n = 40) total, with each tree serving as an independent replicate representing a single whole-plant measurement per trait.

Prior to interpreting model outputs, statistical assumptions were thoroughly checked by visually inspecting model residuals using Quantile–Quantile (Q-Q) plots for normality and Residuals-vs-Fitted plots for linearity and homoscedasticity. Traits were evaluated raw unless diagnostic plots revealed structural non-linearity or heteroscedasticity. Based on these visual diagnostics, six morpho-anatomical traits were natural log-transformed (log(x)) to stabilize variance and satisfy linear model criteria: Leaf Volume (LV), Relative Bark Thickness, Huber Value (HV), Vessel Area (VA), Vessel Density, and Cavitation Index (CVI). All other traits, including Leaf Mass per Area (LMA), Wood Density (WD), Fiber Fraction, Parenchyma Fraction, Vessel Fraction, Stomatal Density, Leaf Chlorophyll, and all flammability metrics (Maximum Temperature, Burnt Biomass, and Burn Time), strictly conformed to parametric assumptions and were analyzed using raw, untransformed values.

For the highly right-skewed trait Leaf Dry Matter Content (LDMC), log-transformation was insufficient to resolve residual non-normality. We therefore analyzed LDMC using a Generalized Linear Model (GLM) with a Gamma distribution and a log-link function. The structural validity of the Gamma GLM was verified using simulated uniform quantile residuals via the DHARMa package in R [65].

All standard linear models and the GLM were executed using Type III Sum of Squares via the Anova function in the car package [66] to ensure robust, unbiased *F*-statistics in the presence of slightly unbalanced group variance. Due to the exploratory nature of our study, and also because the 20 traits capture independent traits and structural organ systems (leaf economics, hydraulics, protection, and combustion), we did not correct for multiple univariate tests. Applying strict multiple-testing adjustments (like FDR or Bonferroni) would drastically inflate our Type II error rate (false negatives) and potentially mask subtle but biologically meaningful signals that we wanted to examine. However, nominal *p*-values are reported to prioritize ecophysiological trends and minimize Type II error rates within this exploratory framework. All statistical analyses were conducted in R Studio (v. 4.6.1) [67].

To visualize functional trait composition and differences within samples and between the two species, we performed a principal component analysis (PCA) ordination using the software PAST 4 [68]. A biplot with trait vectors was plotted to assess the associations between traits and their influence on the ordination. Prior to performing the PCA, the functional trait dataset was screened for mathematical redundancies and compositional dependencies. Stem anatomical traits (fiber, parenchyma, and vessel fractions) quantified via the grid-intersect method represent compositional data subject to a strict constant-sum constraint (totaling 100%). Because vessel fraction occupied a small and relatively stable proportion of the total area, the remaining space represented a direct zero-sum tradeoff between structural and storage tissues, resulting in severe multicollinearity between fiber and parenchyma fractions (Spearman’s rank correlation: r = −0.97, *p* < 0.001). To break this mathematical dependency and avoid artificially distorting the ordination axes, fiber fraction was removed from the PCA matrix, while parenchyma fraction was retained as an indicator of stem storage capacity. Second, mathematically derived traits were excluded from the primary ordination matrix to prevent artificial weighting of specific trait suites. Specifically, leaf volume (derived as Leaf Area × Leaf Thickness) and the cavitation vulnerability index (derived as vessel diameter/vessel density) were omitted because their inclusion would introduce perfect structural dependencies with their respective parent traits. Retaining both parent measurements and their geometric or physiological products would artificially tilt the PCA axes toward those dimensions by double-counting the underlying anatomical investment. Our PCA was therefore run using data of the remaining 17 traits after the appropriate transformations and after standardizing the values from 0 to 1.

To determine whether the segregation of functional strategies between the rainforest and savanna *Grevillea* was statistically significant, we conducted a one-way Permutational Multivariate Analysis of Variance (PERMANOVA; α = 0.05). Prior to analysis, all 17 continuous trait variables were z-score standardized (mean = 0, standard deviation = 1) to account for differing units of measurement and to prevent traits with large absolute values from disproportionately weighting the distance matrix. The PERMANOVA was calculated using a Euclidean distance matrix with 9999 permutations. Because PERMANOVA can be sensitive to differences in within-group variance, we subsequently performed a Homogeneity of Multivariate Dispersions test (PERMDISP) to verify that a significant PERMANOVA reflected a true shift in multivariate centroids rather than a false positive driven by unequal within-group dispersion. All multivariate tests were performed in PAST 4 [68].

## 3. Results

After assessing 20 functional traits to compare our congeneric species, we found significant differences in trait behavior for many of the leaf, stem and anatomical traits between the rainforest and savanna species, but very little divergence in their flammability traits (See Table S1 for model outputs).

### 3.1. Leaf Traits

**Figure 1:**
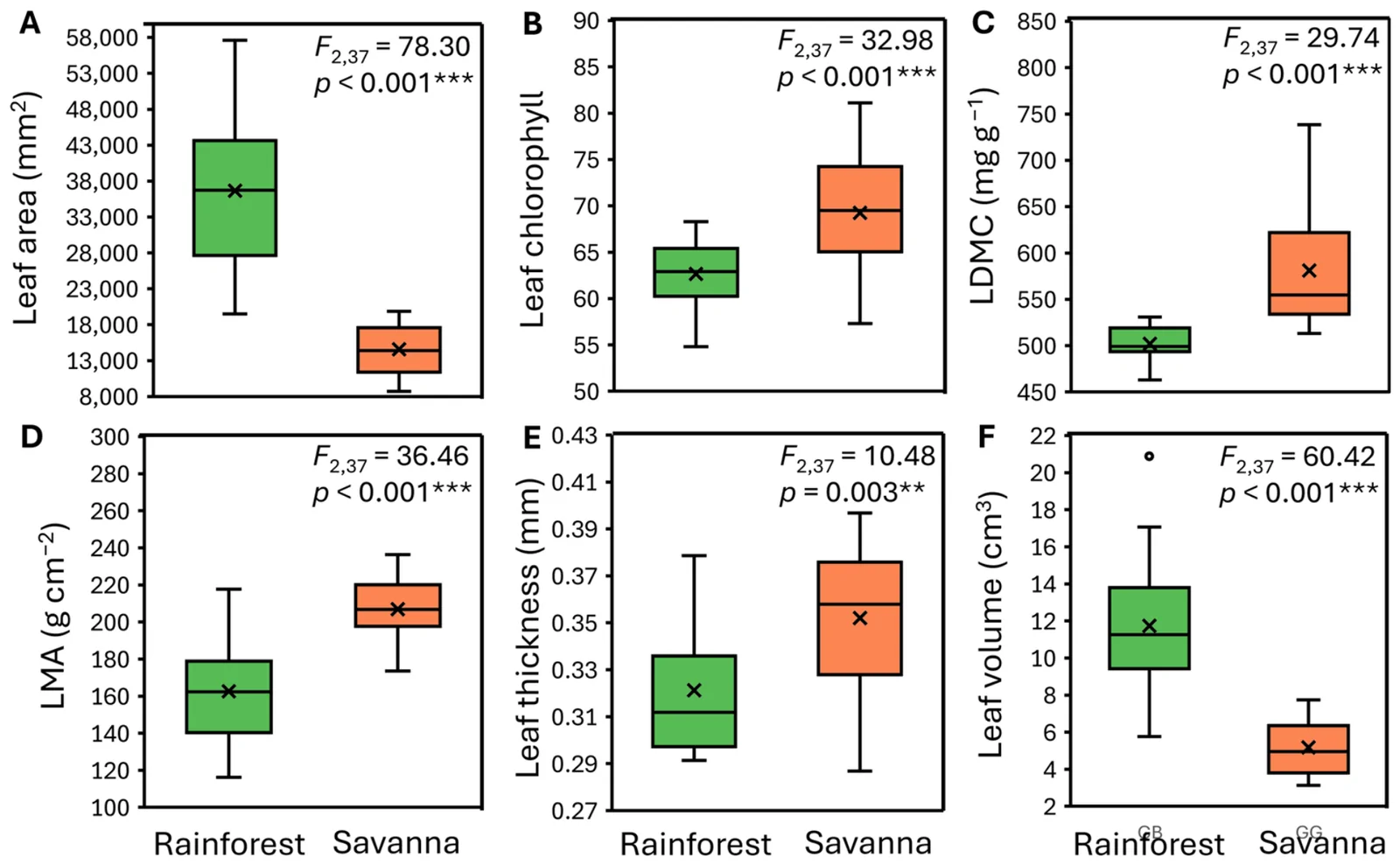
Divergence of leaf functional traits in rainforest and savanna species of *Grevillea* (Proteaceae) (green and orange boxes respectively, with N = 20 samples per species). Boxplots illustrate the differences in trait expression between species for (**A**) leaf area, (**B**) atLEAF chlorophyll content, (**C**) leaf dry matter content (LDMC), (**D**) leaf mass per area (LMA), (**E**) leaf thickness, and (**F**) leaf volume. Boxes include the 25th percentile, median, mean (as a cross), and 75th percentile, while the whiskers indicate the 5th and 95th percentiles. Circles represent outliers. Significance of differences between species is based on analysis of covariance (ANCOVA) models (Table S1), with habitat type included as a fixed effect and tree size (DBH) as a continuous covariate. LDMC was performed using a Generalized Linear Model (GLM) with a Gamma distribution and a log-link function. F-statistics and *p*-values were evaluated using partial (Type II) Sum of Squares via the R car package to isolate habitat effects independently of variation in tree size (*p* < 0.01 **; *p* < 0.001 ***).

All leaf functional traits tested were significantly different between the congeners. The rainforest *Grevillea* demonstrated a greater leaf area (Figure 1A) and leaf volume (Figure 1F) than the savanna species, but lower leaf chlorophyll content (Figure 1B), LDMC (Figure 1C), LMA (Figure 1D), and average leaf thickness (Figure 1E) (see also Table 2 for trait means and S.D.).

### 3.2. Stem Traits

**Figure 2:**
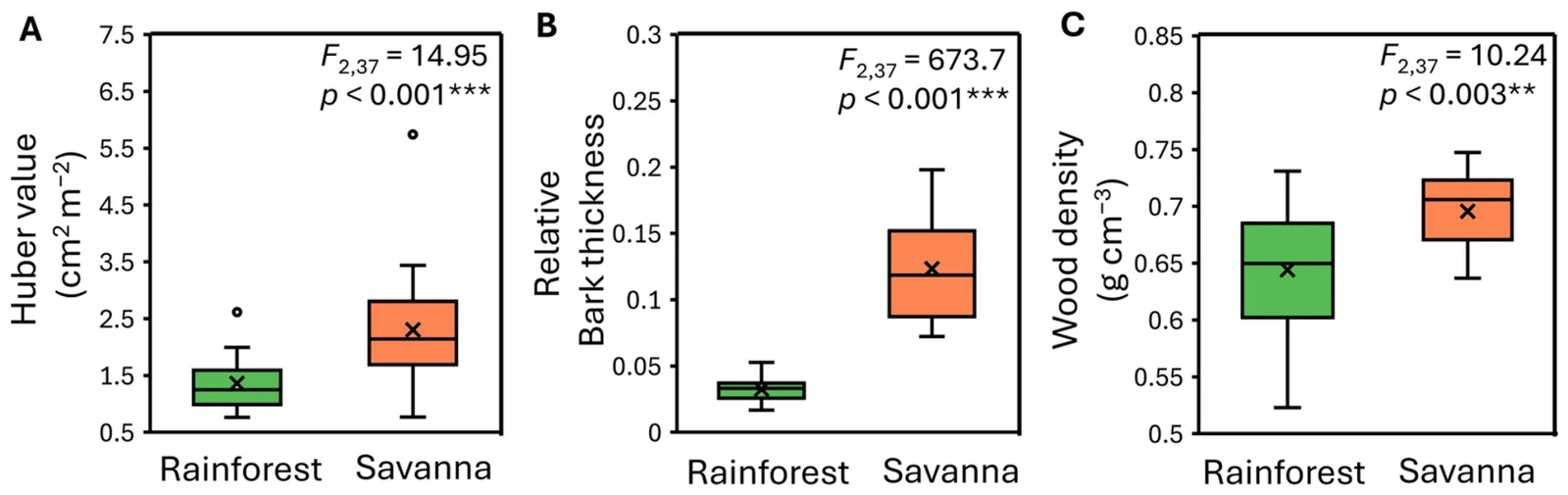
Comparison of wood traits between two congeneric *Grevillea* species (Proteaceae) of the rainforest (*G. baileyana*) and savanna (*G. glauca*) (N = 20 samples for each species). Boxplots represent the range of measures for (**A**) huber values, (**B**) relative bark thickness, and (**C**) wood density and include the 25th percentile, median, mean (as a cross), and 75th percentile, while the whiskers indicate the 5th and 95th percentiles. Circles represent outliers. Significance of differences between species is based on analysis of covariance (ANCOVA) models (Table S1), with habitat type included as a fixed effect and tree size (DBH) as a continuous covariate. F-statistics and *p*-values were evaluated using partial (Type II) Sum of Squares via the R car package to isolate habitat effects independently of variation in tree size (*p* < 0.01 **; *p* < 0.001 ***).

The rainforest *Grevillea* was found to have lower Huber values (Figure 2A), relative bark thickness (Figure 2B) and wood density (Figure 2C) than the savanna *Grevillea*.

### 3.3. Anatomical Traits

**Figure 3:**
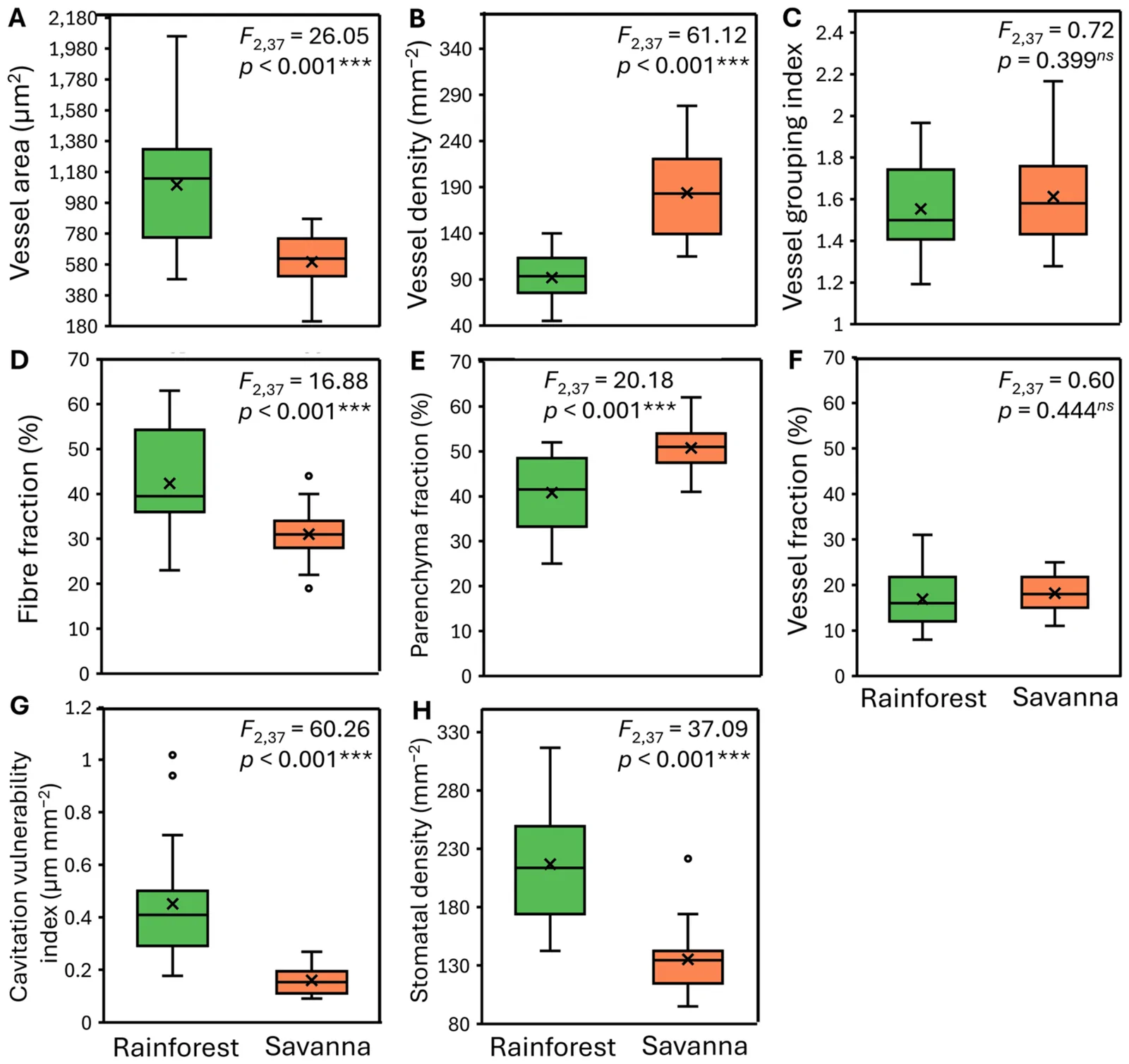
Anatomical trait divergence of convergent rainforest and savanna *Grevillea* species (green and orange boxes respectively, with N = 20 samples per species). Boxplots represent the range of values for the anatomical traits: (**A**) vessel area, (**B**) vessel density, (**C**) vessel grouping index, (**D**) fiber fraction, (**E**) parenchyma fraction, (**F**) vessel fraction, (**G**) cavitation vulnerability index, and (**H**) stomatal density. Boxes include the 25th percentile, median, mean (as a cross), and 75th percentile, while the whiskers indicate the 5th and 95th percentiles. Circles represent outliers. Significance of differences between species is based on analysis of covariance (ANCOVA) models (Table S1), with habitat type included as a fixed effect and tree size (DBH) as a continuous covariate. F-statistics and *p*-values were evaluated using partial (Type II) Sum of Squares via the R car package to isolate habitat effects independently of variation in tree size (*ns* = not significant; *p* < 0.001 ***).

The rainforest *Grevillea* species exhibited greater average vessel area (Figure 3A) along with a lower total vessel density (Figure 3B). The vessel grouping index (Figure 3C) was not significantly different between the congeners. The savanna species was found to contain a lower fiber fraction (Figure 3D) and a higher parenchyma fraction (Figure 3E) than the rainforest species, while vessel fraction showed no significant differences. Lastly, cavitation vulnerability index (Figure 3G) and stomatal density (Figure 3H) were both significantly higher in the rainforest species than in the savanna.

### 3.4. Flammability

**Figure 4:**
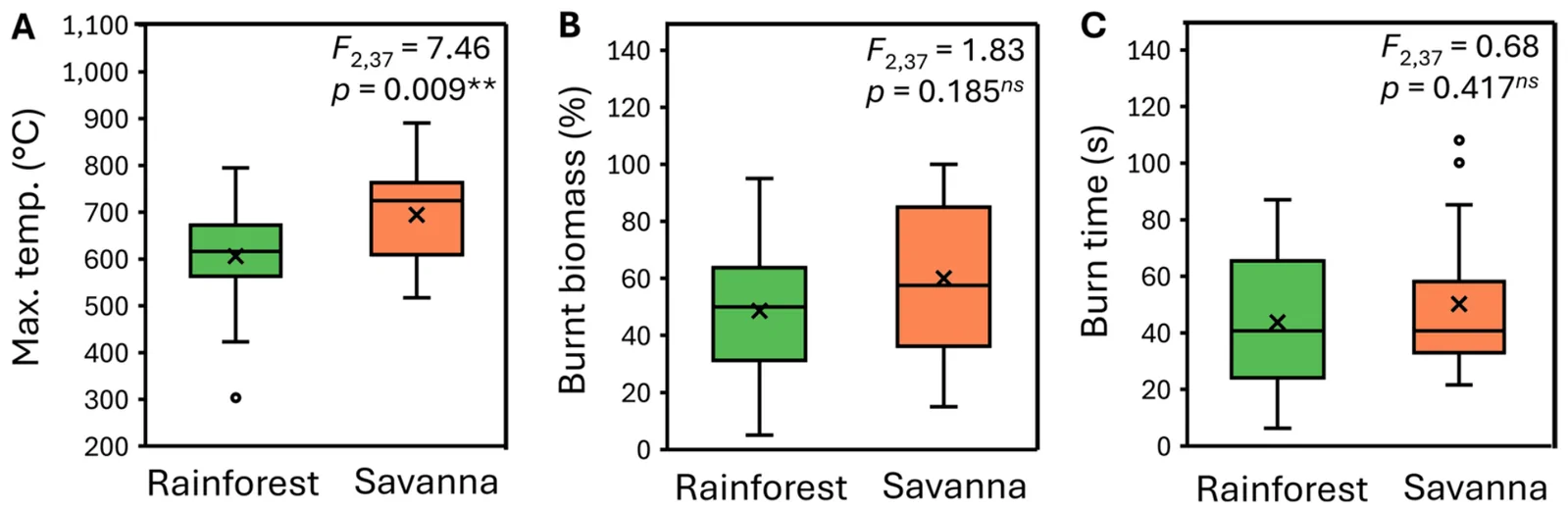
Boxplots comparing flammability measures at the shoot level: (**A**) maximum burn temperature, (**B**) proportion of burnt biomass, and (**C**) burn time of congeneric species of *Grevillea* in rainforest (*G. baileyana*: green boxes) and savanna (*G. glauca*: orange boxes) (N = 20 samples per species). Boxes include the 25th percentile, median, mean (as a cross), and 75th percentile, while the whiskers indicate the 5th and 95th percentiles. Circles represent outliers. Significance of differences between species is based on analysis of covariance (ANCOVA) models (Table S1), with habitat type included as a fixed effect and tree size (DBH) as a continuous covariate. F-statistics and *p*-values were evaluated using partial (Type II) Sum of Squares via the R car package to isolate habitat effects independently of variation in tree size (*ns* = not significant; *p* < 0.01 **).

The savanna *Grevillea* burnt at a significantly higher maximum temperature than its rainforest congener (Figure 4A), but there were no significant differences between the rainforest and savanna species in burn time (Figure 4B) and burnt biomass (Figure 4C).

### 3.5. Multidimensional Trait Space

**Figure 5:**
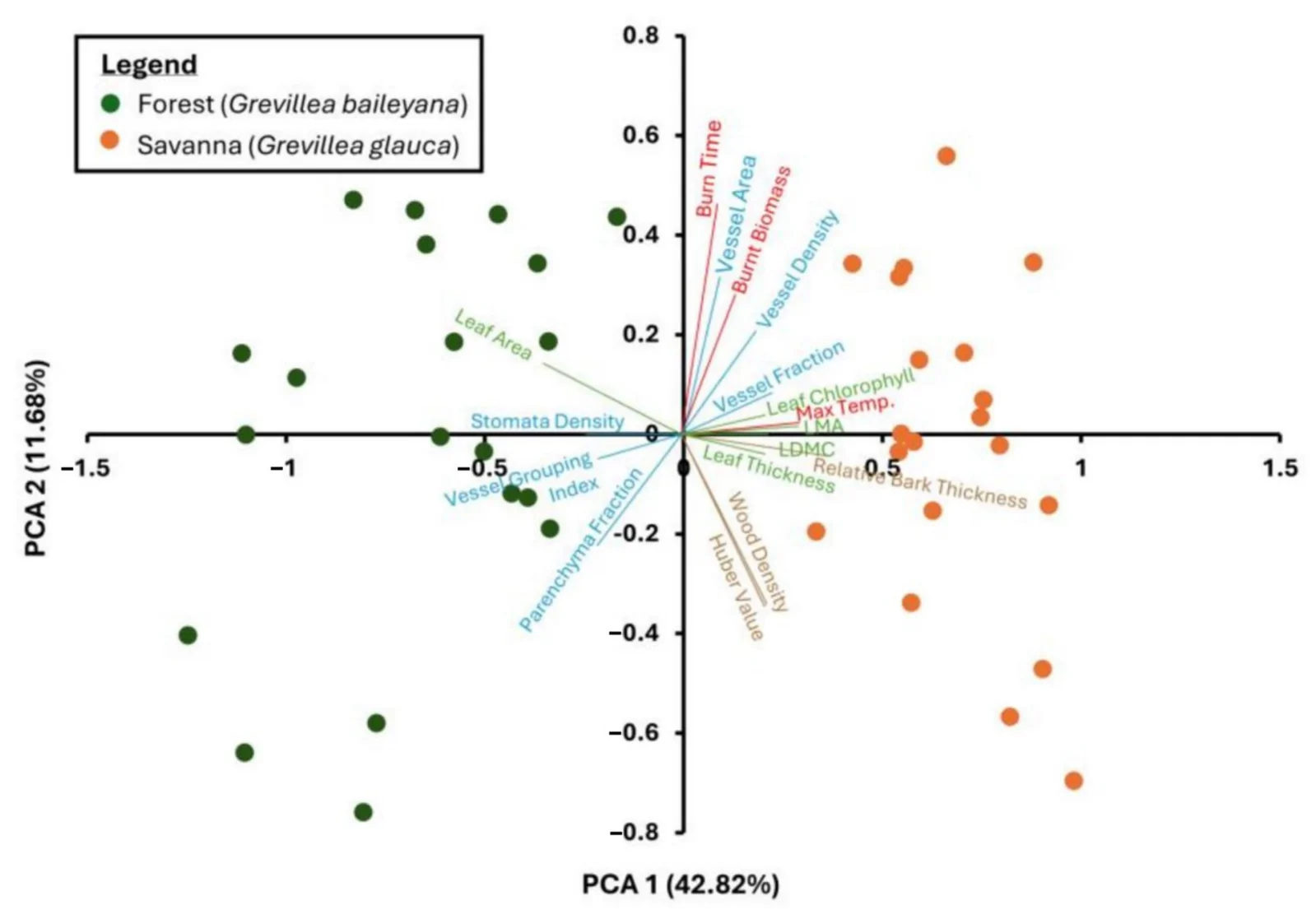
Principal component analysis (PCA) ordination of functional traits measured across 20 replicates of each of the *Grevillea* species, the rainforest *Grevillea baileyana* represented by dark green circles and the savanna *G. glauca* with orange circles. Trait vectors are denoted by lines, the length of which indicates the strength of their influence on the ordination. Leaf traits are represented by green vectors and include leaf area, leaf chlorophyll, leaf mass per area (LMA), leaf dry matter content (LDMC), and leaf thickness. Brown vectors represent the stem traits: Huber value, wood density, and relative bark thickness. Blue vectors signify stem anatomical traits including stomata density, vessel grouping index, parenchyma fraction, vessel area, vessel density, and vessel fraction. Red vectors are flammability traits including burn rate, burn time, burnt biomass, and maximum burn temperature. See Table S2 for PCA loadings.

Our PCA ordination showed a clear segregation between the congeneric rainforest and savanna species samples in terms of their functional trait composition. A PERMANOVA confirmed that this separation was significant (F = 14.85, *p* < 0.001). The two species are distinguished along the PCA 1 axis (42.8% of the total variation), with high values of leaf chlorophyll, LMA, LDMC, and leaf thickness in the savanna species, opposite the lower values of leaf area and leaf volume in the rainforest species (Figure 5). A few anatomical traits are also associated along PCA axis 1, including the opposing savanna species’ high vessel fraction and the rainforest species’ lower stomatal density, VGI, and parenchyma fraction (Figure 5). The second axis (11.7% of the total variation) was related to flammability traits, i.e., burn rate, burn time, and burnt biomass, as well as a few anatomical (vessel area and vessel density) and wood traits (Huber value and wood density). Therefore, these traits are orthogonal to PCA axis 1 and associated traits (i.e., leaf chlorophyll, LMA, LDMC, and leaf thickness) and represent separate or independent functional strategies.

## 4. Discussion

Plant functional trait behaviors are typically associated with environmental filters. To explore how species from divergent habitats may differ in their functional trait behavior, we compared 20 leaf, stem, anatomical, and flammability traits across a pair of congeneric *Grevillea* species, one residing in a rainforest and the other in a savanna environment. Overwhelmingly, the trait divergences between the congeneric pair align with our overarching hypothesis of habitat being the key driver of many predicted trait behaviors. However, three out of the 20 traits compared exhibited the opposite effect, while four traits, notably some related to flammability, did not exhibit significant differences.

### 4.1. Leaf Traits

Our findings on leaf traits were mostly in agreement with our predictions. Leaf area was greater in the rainforest *Grevillea*, which likely reflects a higher rate of photosynthesis and a high growth rate [69]. This would be important in a light-limited environment such as in the rainforest *Grevillea* to reach the canopy faster. Conversely, in the savanna species, it is more advantageous for trees to grow more slowly and to economize on water use and nutrients they get as efficiently as possible. Thus, we found that the savanna species have a higher LMA and leaf thickness compared to the rainforest species [33], which may reflect strategies for resisting water loss.

Leaf chlorophyll content was one trait for which we were unsure of the directional response. On the one hand, we may hypothesize that leaf chlorophyll content would be higher in the rainforest species because rainforest plants need to maximize light capture in an environment where light is a limiting resource [70]. Yet, we found the opposite pattern in our congeners. The significantly higher area-based chlorophyll content observed in the savanna species may be coupled with its structural leaf adaptations to an open, high-irradiance habitat. Savanna species frequently exhibit higher leaf mass per area (LMA) than their rainforest counterparts [33], and this was the case for our savanna *Grevillea*. A higher LMA could result in packing denser cellular machinery into a given surface area [71], thereby explaining our observations.

### 4.2. Stem Traits

For our stem traits, the mean wood density of the savanna *Grevillea* was significantly higher than that of the rainforest *Grevillea*. However, relative bark thickness (BT) and Huber value differed significantly and in alignment with our predictions. We hypothesized that the savanna species would have a higher relative BT [46], while the forest species may not require such a large investment in bark to protect themselves from fire [72] because of the lack of fire disturbances in the rainforest. Follow-up studies could compare bark density or other bark functional traits [73] to examine how these traits are subjected to environmental filtering.

The higher Huber value of the savanna relative to the rainforest species is in agreement with our initial prediction and is likely a habitat-associated response to cope with a higher rate of transpiration during the dry season. In tandem with their higher parenchyma fraction, it is likely that the savanna species invests more in sapwood area as a water storage mechanism, and similar findings have been made when comparing dry forest and wet forest trees [26].

### 4.3. Anatomical Traits

Six of the eight anatomical traits examined differed significantly between species, while the direction of the effect of five of the traits, including stomatal density, vessel area, vessel density, cavitation vulnerability index, and parenchyma fraction, aligned with our original predictions. Our initial hypotheses were driven largely by the understanding that forest species depend on a greater efficiency of water transport and gas exchange due to the high rates of precipitation [48,52]. These environmental factors correlate with an observed higher vessel area and stomatal density, respectively, in the forest species of *Grevillea*.

In tandem with larger vessel area and higher vessel grouping, we also found that the forest *Grevillea* had a greater cavitation vulnerability index, which agrees with its habitat setting [50]. Notably, we found no significant difference in vessel fraction between *G. baileyana* and *G. glauca*.

We identified contrasting patterns in fiber fraction and parenchyma fraction of the rainforest and savanna species, which we speculate is due in part to strong environmental filtering. Previous studies have documented higher parenchyma fractions in woody plants from dry environments [28,32,74]. However, the higher fiber fraction we observed in the rainforest species was not in agreement with our original hypothesis. Yet, the explanation for this could be quite straightforward. Additionally, the tradeoffs between the vessel, parenchyma, and fiber fractions may also play a role. Since the vessel fraction is similar between the two species and the parenchyma fraction is higher in the savanna species for water storage, the fiber fraction might in part be lower in the savanna species because of the space tradeoff within the sapwood [75]. A deeper look at ultrastructural components of fiber wall thickness [29] may also reveal a further mechanistic dimension in this tradeoff.

Lastly, we were surprised to find no significant difference in vessel grouping indices between the congeners, given that we have observed distinct differences in wet and dry forest species in a previous study [26]. It could be that the relationship may not hold up for all congeneric taxa from divergent environments, but more extensive sampling across taxa will be required to parse out the patterns and mechanisms of this trait as a response to environmental conditions.

### 4.4. Flammability

As a family, the Proteaceae are flammable in general [22], and our data is consistent with this finding. Still, we found that the savanna species burnt at a higher maximum temperature, which is consistent with their higher leaf dry matter content [20,22]. However, the reasons for the lack of differences in burnt biomass and burn time between the congeners are unclear. In follow-up studies, it may be beneficial to explore leaf-level flammability and chemistry. We also acknowledge that while the stove-top burning trials may provide a standardized measure of flammability, it may not fully represent fire behavior under natural field conditions. Therefore, future studies examining how whole plant-level architecture affects flammability will be instructive [20,63].

### 4.5. Intraspecific Trait Variation and Study Limitations

In efforts to understand the level of variation in traits specific to each *Grevillea* population, it is worth making a few descriptive observations of intraspecific variation in both study species within their respective populations. We would expect the rainforest *Grevillea* to demonstrate a lower overall variation in any given functional trait because a rainforest environment is generally considered a more climatically buffered habitat relative to a savanna [76]. Instead, we found that the coefficients of variation (CV) varied greatly depending on traits. For instance, we observed high CVs in leaf area in the rainforest *Grevillea* compared to the savanna *Grevillea*. This may reflect higher morphological plasticity of leaves in the rainforest species because of greater heterogeneity in light environments and competition for sunlight even among leaves in the canopy [77]. Conversely, less competition in the savanna’s open canopy could additionally explain its observed lower variability in leaf area.

We caution that our findings should be interpreted with care because we were only able to study trait behavior of the congeneric *Grevillea* pair at a single population. Nevertheless, we speculate that many of the trait behaviors observed are consistent with environmental filtering because the two species do not overlap in habitat. Sampling these and other forest–savanna congeners across a broader geographical range will deepen our understanding of the intraspecific variation or phylogenetic constraints in functional traits [15,26]. This is especially true for harder-to-sample functional traits such as those pertaining to the wood anatomical economics spectrum [25,78].

## 5. Conclusions

Studying congeneric species and their functional traits allows for a deeper understanding of habitat-associated trait strategies. In turn, this may allow us to better predict how species will respond to climatic extremes. The contrasting habitats occupied by our congeneric *Grevillea* pair (forest vs. savanna) may have played a key role in the observed divergences in leaf, wood and anatomical traits, but the two species appear to share a similar magnitude of shoot-level flammability. However, broader sampling of these species across their geographical ranges may yield further insights into intraspecific trait variation. In general, many of the traits in the rainforest species reflected acquisitive strategies, while those in the savanna species were more indicative of resource-conservation strategies. Our research highlights the need for more congeneric pair studies to examine the nuances of functional trait variation, with an emphasis on less commonly studied anatomical traits such as wood and leaf anatomy.

## Figures and Tables

**Table 1:** Premises and predictions of leaf, stem and anatomical functional trait behaviors in species occurring in rainforest and savanna environments.

Trait	Units	Predicted Effect	Premise	References
***Leaf traits***				
Leaf Area (LA)	mm^2^	*Forest > Savanna*	The forest species will have larger leaf areas to enable higher light capture in a more productive and crowded environment while not being constrained by evapotranspiration.	Cornwell and Ackerly [41]; Tng et al. [33]
Leaf chlorophyll content	unitless	Unknown	Unknown effect	
Leaf Dry Matter Content (LDMC)	mg g^−1^	*Forest < Savanna*	High LDMC is associated with exposure to less precipitation and thicker leaves that are found within species located in the savanna.	Hu et al. [42]
Leaf Mass per Area (LMA)	g cm^−2^	*Forest < Savanna*	The savanna species will have a greater LMA because the leaves require less water under dry conditions, are more rigid, and are more resistant to wilting.	Cornwell and Ackerly [41]; Pooter et al. [43]
Leaf Thickness (LT)	mm	*Forest < Savanna*	The savanna species will have a larger thickness because plants in regions with higher annual temperatures tend to have thicker leaves.	Prior et al. [44]
Leaf Volume (LV)	mm^3^	Unknown	Unknown effect	
***Stem traits***				
Huber Value (HV)	cm^2^ m^−2^	*Forest < Savanna*	The savanna species will have a higher Huber value. The savanna species will invest more sapwood area per unit of leaf area to cope with the high transpiration rates during the dry season.	Gotsch et al. [45]
Relative Bark Thickness	unitless	*Forest < Savanna*	The savanna species have a greater relative bark thickness to adapt to higher fire frequencies and confer greater protection to the cambium.	Lawes et al. [46]; Tng et al. [33]
Wood Density (WD)	g cm^−3^	*Forest < Savanna*	The savanna species will have a denser wood, consistent with slower tree growth.	Kraft et al. [47]; Tng et al. [33]
***Anatomical traits***				
Vessel Area (VA)	μm^2^	*Forest > Savanna*	The forest species will have a greater vessel area because larger vessels are associated with higher water conductivity, as expected in forest species. Also, vessel sizes are correlated with the length of the stem, and rainforest species tend to be longer-stemmed.	Apgaua et al. [26,32]; Fajardo et al. [48]
Vessel Grouping Index (VGI)	unitless	*Forest > Savanna*	The forest species will have a higher vessel grouping index because vessel grouping may aid in increased water conductivity expected for forest trees, whereas solitary vessels may be hydraulically safer for savanna species	Carlquist [49]; Apgaua et al. [26]
Vessel Density (Vden)	mm^−2^	*Forest < Savanna*	The forest species will have a lower average vessel density due to a tradeoff between large vessels and hydraulic safety.	Apgaua et al. [26,32]
Cavitation Vulnerability Index (CVI)	unitless	*Forest > Savanna*	The forest will have a higher cavitation vulnerability index than savanna species since they can be expected to be more drought-sensitive.	Carlquist [50]
Vessel Fraction	%	*Forest > Savanna*	The forest species will have a greater vessel fraction because the number of vessels is correlated with the length of the stem and the water conductivity rate of the species.	Fajardo et al. [48]
Parenchyma Fraction	%	*Forest < Savanna*	The savanna species will have a higher parenchyma fraction because the savanna has periodic precipitation cycles where the species needs to store their water intake within the parenchyma cells.	Zhang et al. [51]
Fiber Fraction	%	*Forest < Savanna*	Savanna species should have higher fiber fractions, as they will invest more in structural support than forest species due to the more exposed and disturbance-prone environment they occur in.	Ziemińska et al. [29]
Stomatal density	mm^−2^	*Forest > Savanna*	The forest species will have a higher stomatal density to conduct a greater rate of gas exchange in comparison to the savanna species.	Wu et al. [52]
***Flammability***				
Maximum Temperature	°C	*Forest < Savanna*	The savanna species will have a greater maximum burn temperature because the species occurs in a drier environment that is more prone to fire disturbances.	Ortiz et al. [20]
Burnt Biomass	%	*Forest < Savanna*	The savanna species will have a greater proportion of burnt biomass due to the dry climatic conditions of the savanna and the region’s need for fire.	Ortiz et al. [20]
Burn Time	seconds	*Forest < Savanna*	The savanna species will have a longer burn time because the species occurs in a drier environment and is prone to fire disturbances.	Ortiz et al. [20]

**Table 2:** Means, standard deviations (SD), and coefficients of variation (CV) of each trait measured for a congeneric pair of *Grevillea* species in the rainforest and the savanna.

Trait	Rainforest (*Grevillea baileyana*) (n = 20)			Savanna (*Grevillea glauca*) (n = 20)		
	Mean	SD	CV	Mean	SD	CV
*Leaf traits*						
Leaf Area (mm^2^)	36,645.632	10,666.534	0.291	14,578.847	3288.364	0.226
Chlorophyll Content	61.783	2.888	0.047	68.524	4.362	0.064
Leaf Dry Matter Content (mg g^−1^)	502.133	17.621	0.035	581.097	65.710	0.113
Leaf Mass per Area (g cm^−2^)	162.597	27.551	0.169	206.901	17.073	0.083
Leaf Thickness (mm)	0.321	0.027	0.085	0.352	0.032	0.091
Leaf Volume (cm^3^)	11.744	3.495	0.298	5.180	1.466	0.283
*Stem traits*						
Huber Value (cm^2^ m^−2^)	1.359	0.481	0.354	2.303	1.077	0.468
Relative Bark Thickness	0.649	0.188	0.290	2.467	0.762	0.309
Wood Density (g cm^−3^)	0.649	0.052	0.080	0.692	0.029	0.041
*Anatomical traits*						
Vessel Area (µm^2^)	1096.091	0.000	0.361	597.57	0.000	0.283
Vessel Density (mm^−2^)	92.016	25.723	0.280	183.732	46.819	0.255
Vessel Grouping Index	1.554	0.202	0.130	1.612	0.224	0.139
Fiber Fraction (%)	42.35	10.66	0.25	31.05	5.871	0.189
Parenchyma Fraction (%)	40.80	8.07	0.20	50.75	5.514	0.109
Vessel Fraction (%)	16.90	6.28	0.37	18.2	4.060	0.223
Vulnerability Index (μm mm^−2^)	0.447	0.216	0.484	0.158	0.056	0.356
Stomata Density (mm^−2^)	216.843	50.079	0.231	135.329	31.436	0.232
*Flammability*						
Max Temp (°C)	606.110	109.511	0.181	694.255	92.922	0.134
Burnt Biomass (%)	48.650	25.004	0.514	60.000	27.815	0.464
Burn Time (s)	43.706	24.249	0.555	50.160	24.838	0.495

## Data Availability

The original contributions presented in this study are included in the article. Further inquiries can be directed to the corresponding author.

## Supplementary Materials

The following supporting information can be downloaded at https://www.mdpi.com/article/10.3390/biology15212031/s1. Table S1: Analysis of Covariance (ANCOVA) summary of habitat effects on plant functional traits, with stem diameter (DBH) included as a continuous covariate; Table S2: PCA loadings for the 17 traits used.
