# Supplementary material for: Divergent Leaf and Stem Traits but Similar Flammability in Two Contrasting Tropical Grevillea Species
Source: Biology (Basel). 2026 Nov 7;15(21):2031. doi: 10.3390/biology15212031 (PMC13762354; doi:10.3390/biology15212031)
Supplement: Supplementary file 1 [file biology-15-02031-s001.zip › biology-4606138-supplementary.pdf]

Supplementary Material for Tng et al. **Divergent leaf and stem traits but similar flammability in two contrasting tropical *Grevillea* species**

Table S1. Analysis of Covariance (ANCOVA) summary of habitat effects on plant functional traits, with stem diameter (DBH) included as a continuous covariate. F-statistics and p-values were evaluated using partial (Type II) Sum of Squares via the R car package to ensure the main effect of habitat is evaluated independently of variation in tree size. Residual degrees of freedom was 37 for all models. Significant effects ($p < 0.05$) are highlighted in bold.

Trait	Transformation	Habitat (F-value)	Habitat (p)	DBH	DBH (p)
Leaf Area (mm^2)	none	**78.296**	**<0.001*** **	1.062	0.269
Leaf Chlorophyll Content	none	**32.983**	**<0.001*** **	0.743	0.394
Leaf Dry Matter Content (mg/g)	none	**29.738**	**<0.001*** **	0.183	0.671
Leaf Mass per Area (g/m^2)	none	**36.463**	**<0.001*** **	0.079	0.781
Leaf Thickness (mm)	none	**10.480**	**0.003** **	0.028	0.869
Leaf Volume (cm^3)	log(x)	**60.416**	**<0.001*** **	1.252	0.27
Relative Bark Thickness (cm^2)	log(x)	**673.7**	**<0.001*** **	**104.5**	**<0.001*** **
Huber Value	log(x)	**14.951**	**<0.001*** **	0.028	0.867
Wood Dens (g/cm^3)	none	**10.240**	**0.003** **	0.001	0.976
Stomata Dens (mm^{-2})	none	**37.092**	**<0.001*** **	0.082	0.776
Fiber Fraction (%)	none	**16.88**	**<0.001*** **	0.193	0.663
Parenchyma Fraction (%)	none	**20.18**	**<0.001*** **	<0.001	0.997
Vessel Fraction (%)	none	0.598	0.444	0.615	0.438
Vessel Area (mm^2)	log(x)	**26.047**	**<0.001*** **	0.817	0.372
Vessel Dens (mm^{-2})	log(x)	**61.122**	**<0.001*** **	0.703	0.407
Vessel Grouping Index	none	0.727	0.399	0.090	0.765

Cavitation Vuln Index (μm/mm^2)	log(x)	**60.263**	**<0.001*** **	0.037	0.848
Max Temp (°C)	none	**7.467**	**0.009*** **	0.664	0.420
Burnt Biomass (%)	none	1.827	0.185	0.691	0.411
Burn Time (s)	none	0.675	0.417	0.097	0.758

Table S2. PCA loadings for the 17 traits used.

	PC 1	PC 2	PC 3
Leaf Dry Matter Content	0.244	-0.003	-0.325
Leaf Area	-0.323	0.164	0.166
Leaf Mass per Area	0.288	-0.076	0.210
Leaf thickness	0.181	-0.110	0.396
Leaf chlorophyll	0.244	0.058	-0.177
Stomatal density	-0.236	-0.037	0.315
Relative Bark thickness	0.321	-0.072	-0.127
Huber value	0.238	-0.413	0.087
Wood densiry	0.238	-0.413	0.087
Parenchyma Fraction	-0.270	-0.218	-0.307
Vessel fraction	0.268	0.079	0.060
Vessel area	0.116	0.296	0.516
Vessel grouping index	-0.271	-0.056	0.191
Vessel density	0.053	0.113	0.189
Max. temp.	0.311	0.022	0.160
Burnt Biomass	0.206	0.403	-0.182
Burn time	0.126	0.533	-0.092
